# No Change in Social Decision-Making Following Transcranial Direct Current Stimulation of the Right Temporoparietal Junction

**DOI:** 10.3389/fnins.2018.00258

**Published:** 2018-04-19

**Authors:** Laura F. Blair-West, Kate E. Hoy, Phillip J. Hall, Paul B. Fitzgerald, Bernadette M. Fitzgibbon

**Affiliations:** ^1^Monash Alfred Psychiatry Research Centre, The Alfred and Monash University Central Clinical School, Melbourne, VIC, Australia; ^2^Epworth Clinic, Epworth Healthcare, Camberwell, VIC, Australia

**Keywords:** right temporoparietal junction, social decision-making, transcranial direct current stimulation, mentalizing, Ultimatum Game, altruistic punishment

## Abstract

The right temporoparietal junction (rTPJ) is thought to play an important role in social cognition and pro-social decision-making. One way to explore this link is through the use of transcranial direct current stimulation (tDCS), a non-invasive brain stimulation method that is able to modulate cortical activity. The aim of this research was therefore to determine whether anodal tDCS to the rTPJ altered response to a social decision-making task. In this study, 34 healthy volunteers participated in a single-center, double-blinded, sham-controlled crossover design. Subjects received 20 min of active/sham anodal tDCS to the rTPJ before undertaking the Ultimatum Game (UG), a neuroeconomics paradigm in which participants are forced to choose between monetary reward and punishing an opponent's unfairness. Contrary to expectations, we found no significant difference between anodal and sham stimulation with regard to either the total number or reaction time of unfair offer rejections in the UG. This study draws attention to methodological issues in tDCS studies of the rTPJ, and highlights the complexity of social decision-making in the UG.

## Introduction

The right temporoparietal junction (rTPJ) is a region of increasing interest in studies of the social brain. It encompasses the supramarginal gyrus, caudal parts of the superior temporal gyrus, and dorsal-rostral parts of the occipital gyrus, and is reciprocally connected to the right prefrontal cortex and temporal lobe (Decety and Lamm, 2007). Functionally, the rTPJ has been implicated in cognitive empathy, the intellectual ability to understand another's state of mind (Shamay-Tsoory, 2011). Specifically, the rTPJ is thought to be involved in the mentalizing process, in which one discerns the mental states of other humans (Frith and Frith, 2006). Using “Theory of Mind” (Frith and Frith, 2005), this information is integrated into a single coherent model used to predict and explain another's behavior and experiences (Saxe and Kanwisher, 2003; Saxe and Wexler, 2005; Vollm et al., 2006). Through its role in empathy processing, rTPJ function may encourage pro-social behavior, and to date functional brain imaging techniques have provided partial evidence for this claim. For example, increased gray matter volume of the rTPJ is linked with altruism (Morishima et al., 2012), and rTPJ activity is heightened during pro-social decision-making (Zanon et al., 2014).

One way to interrogate the link between rTPJ activity and social decision-making is through non-invasive brain stimulation (NIBS) techniques such as transcranial magnetic stimulation (TMS) and transcranial direct current stimulation (tDCS). tDCS has the ability to temporarily modulate neuronal excitability and thus function (Nitsche and Paulus, 2000; Pellicciari et al., 2013; Lauro et al., 2014; Pisoni et al., 2017). In this method, electrodes are applied over the brain region of interest and a reference location, through which low voltage electrical currents are applied via an external, battery-operated stimulator (Dayan et al., 2013). Anodal tDCS is thought to enhance intrinsic neuronal activity by depolarizing resting membrane potentials and increasing the likelihood that neurons will fire. Cathodal tDCS is believed to do the opposite, hyperpolarizing membrane potentials and generally (although not consistently) reducing the likelihood of neuronal firing (Dayan et al., 2013). Of particular utility for sham-controlled studies, a placebo version of tDCS is available and essentially indistinguishable from real stimulation (Nitsche et al., 2008). In a 2012 study applying tDCS to the rTPJ, anodal stimulation improved participants' perspective taking and agency discrimination skills, but mentalizing ability was surprisingly unaffected (Santiesteban et al., 2012). More recently, TMS has been used to provide casual evidence for the role of the rTPJ in mentalizing and pro-social decision-making by incorporating tasks from neuroeconomics (Soutschek et al., 2016; Hill et al., 2017).

NIBS studies typically assess the behavioral impact of stimulation on a relevant task. Neuroeconomics paradigms such as the Ultimatum Game (UG) are commonly used to replicate the nuances of social decision-making in an experimental setting (Lee, 2008). In the UG (Güth et al., 1982), two players must interact to split a set sum of money between themselves. The “proposer” decides upon a division of the available wealth, while the “responder” can choose to either accept or reject this offer. If an offer is accepted then the funds are allocated as was proposed. However, if rejected then neither party receives any money. The outcomes of simulated social tasks such as the UG are often startlingly different to those predicted through “Game Theory,” the mathematical study of strategic decision-making models between rational, intelligent, self-interested parties (Lee, 2008). In the UG, whilst economically advantageous for a proposing player to offer the minimum amount allowed, and the responding player to accept any deal, participants typically decline those which perceived as unfair (Polezzi et al., 2008), even at very high stakes (Cameron, 1999). In fact, mean proposals are typically around 40% of the available sum, and around half of responders reject offers of 30% or less (Güth et al., 1982; Polezzi et al., 2008).

Functional brain imaging reveals complexity in social decision-making during the UG. Unfair offers elicit activity in the anterior insula (AI) and dorsolateral prefrontal cortex (DLPFC), indicating negative emotional arousal and cognitive processing respectively (Rilling et al., 2008). The magnitude of AI response is proportional to the degree of perceived unfairness, and predictive of offer rejection. Opposing action in the DLPFC appears to exert top-down cognitive control over an arguably irrational impulse to reject unfair offers (Rilling et al., 2008). Simultaneously, unfair offers are associated with rTPJ activation, understood to represent mentalizing processes that help to determine an opponent's mental state and intentions (Rilling et al., 2004; Halko et al., 2009; Guo et al., 2013; Van Den Bos et al., 2014). Clearly, refusal of an unfair offer in the UG can result from anger and spite triggered by poor treatment on a personal level (Sanfey et al., 2003). Alternatively however, and of possible relevance to rTPJ function, rejection of an unfair offer in the UG has been interpreted as “altruistic punishment,” a pro-social behavior whereby one forgoes personal needs for the benefit of another (Fehr and Fischbacher, 2003). Altruistic punishment appears to benefit the wider community through the enforcement established social norms (Fehr and Gachter, 2002; Fehr and Fischbacher, 2003), in the case of the UG by encouraging proposers to make fairer offers against future partners. To date, NIBS techniques have been used successfully to disrupt the right DLPFC and decrease rates of unfair offer rejection during UG gameplay (Knoch et al., 2006, 2008), but the rTPJ has not been targeted in this setting.

In the current study, we applied anodal tDCS to the rTPJ prior to participants undertaking the UG to explore the link between rTPJ function and social decision-making. It was hypothesized that anodal stimulation of the rTPJ would enhance mentalizing ability and consequently awareness of an opponent's unfair intentions, thus leading to an increase in pro-social decision-making when compared to sham stimulation. Given that punishing unfairness in the UG may represent altruistic punishment (a pro-social behavior), this effect was expected to take the form of an increase in either the total number of unfair offer rejections in the UG, or a decrease in the response reaction time of unfair offer rejections.

## Materials and methods

### Design

The study was devised as a single-center, double-blinded, crossover, sham-controlled experiment. Participants attended two experimental sessions where they received tDCS before engaging in a social decision-making task. Sessions were separated by at least 72 h to prevent carry-over effects, and the order of stimulation condition applied at each session was counterbalanced between participants.

### Participants

Thirty-four healthy adults were enlisted for the study, comprising 14 males and 20 females, with an age range of 18–48 years (mean 25.14, standard deviation 6.85). All selected participants were right-handed. Participants were excluded if suffering from a serious medical condition, diagnosed with a neurological or psychiatric illness, taking any psychoactive substances, were pregnant, or had non-dental metalwork inside the head or body (i.e., cardiac pacemaker).

Participants were recruited from the general population and provided written consent prior to commencement of the study. The project received ethics approval from the Monash University Human Research and Alfred Ethics Committees.

### Procedure

**Figure 1:**
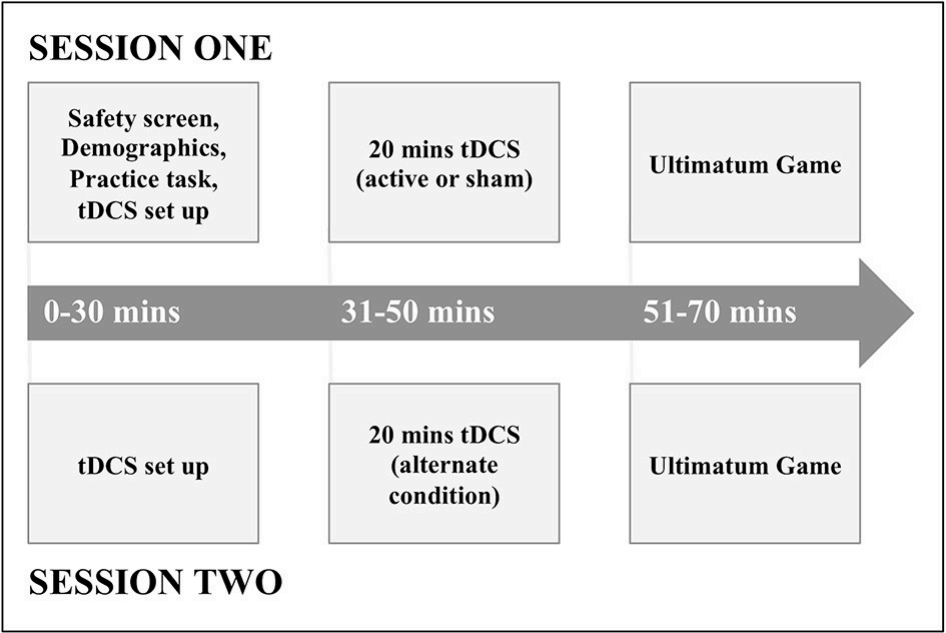
The experimental protocol.

At the beginning of the first session, participants gave consent, basic demographic information, and completed a tDCS safety screen (experimental procedure depicted in Figure 1). Participants were then given a verbal explanation of the UG and played a shortened practice version of the task.

Following this, participants underwent either active or sham anodal tDCS to the rTPJ, before playing the UG immediately afterwards. In a second session at least 72 h later, participants then underwent the alternate condition (active or sham anodal tDCS) to session one, before playing the UG again.

In order for establishment of the double-blind protocol, an independent researcher assigned 5-digit stimulation codes corresponding to active or sham stimulation. These codes would determine the nature of each session conducted by the Eldith Stimulator, ensuring blinding of both the subject and investigator.

### Materials

#### Transcranial direct current stimulation

tDCS was delivered via a battery-operated, constant current Eldith DC stimulator (Model: 0008, Serial: 0083). The anode was placed over CP6 and the cathode over the vertex (Santiesteban et al., 2012), using the 10/20 international system for electroencephalography (EEG) electrode placement (Jasper, 1958). The electrodes were contained within saline soaked sponges.

During the active condition, an electrical current of 1 mA was applied for 20 min via 35 cm^2^ electrodes (current density of 0.029 mA/cm^2^), with 60-s fade-in and 30-s fade-out periods. In the sham condition, stimulation was applied for only 30 s following the 60-s fade in, and then faded out over 30 s. This form of sham stimulation mimics the initial itching and tingling sensations associated with tDCS without provoking the lasting biological effects associated with continued stimulation. This protocol has reliably been shown to be indistinguishable from active stimulation (Gandiga et al., 2006).

**Figure 2:**
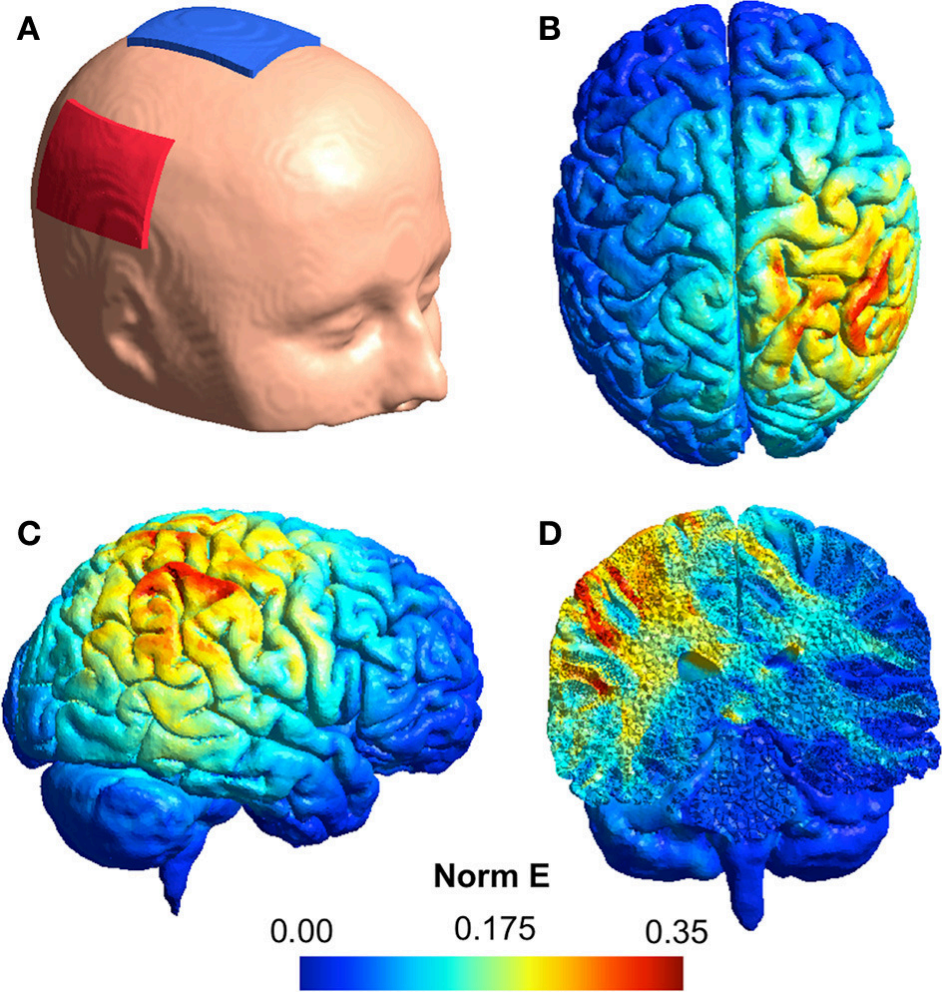
**(A)** An illustration of the tDCS electrode montage with the anode (red) over the rTPJ and the cathode (blue) over the vertex. **(B–D)** Simulation of the normalized electric field distribution in the brain: **(B)** superior view, **(C)** lateral view, **(D)** coronal view.

Simulation of electric field distributions in the brain (shown in Figure 2) was performed using the SimNIBS software (Thielscher et al., 2015) incorporating the template head model included with the software. Models were derived using a current strength of 1 mA applied through 1 mm thick rectangular 5 × 7 cm rubber electrodes featuring rectangular connectors and encased in 3 mm thick sponges. The following default biological tissue conductivity values were used: white matter: 0.126 S/m, gray matter: 0.275 S/m, cerebrospinal fluid: 1.654 S/m, bone: 0.010 S/m, scalp: 0.465 S/m.

#### The ultimatum game

**Figure 3:**
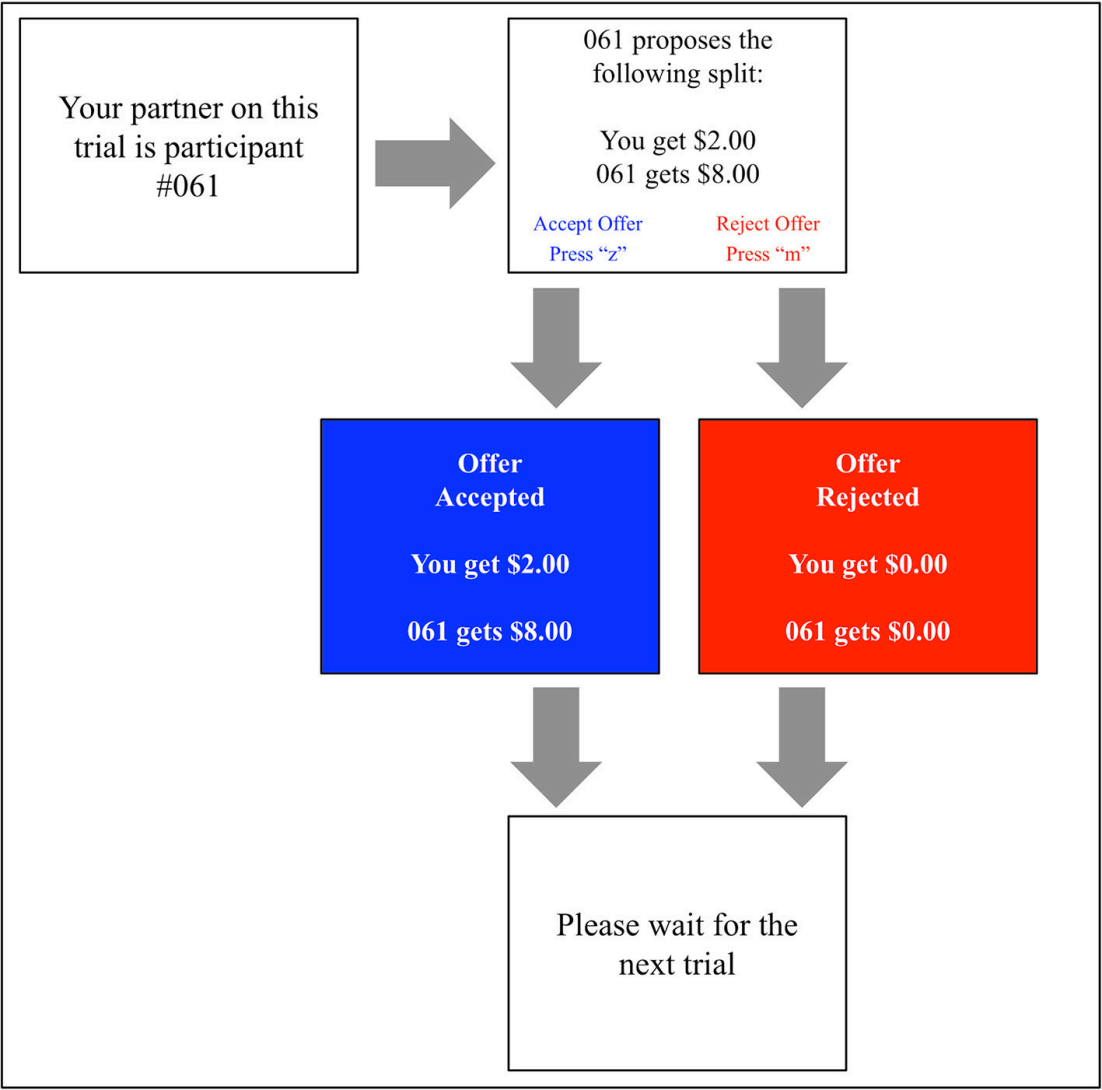
A visualization of the social decision-making task.

A computerized version of the UG (shown in Figure 3) was developed using E-Prime software (Version: 2.0 SP1, Build: 2.0.10.353). The task block comprised 100 identical computer-generated proposals containing 40 fair ($4 or $5 out of 10) and 60 unfair ($1, $2, or $3) offers. This classification of fair vs. unfair offers is in keeping with several other prior studies (Koenigs and Tranel, 2007; Calvillo and Burgeno, 2015).

Each offer was displayed to the participant alongside the identification number of the proposer. Pressing the “z” score on the keyboard allowed the participant to accept this offer, whilst pressing the “m” key prompted its rejection. Each interaction was followed by a visual display reinforcing its outcome (e.g., “#207 got $7. You got $3” or “#207 got $0. You got $0”).

In order to best simulate a genuine social interaction, participants were lead to believe that prior study participants had generated these offers. To increase the believability of this ruse, participants were at initially asked to record 20 offers in the “proposer” role, supposedly to be put to future participants.

### Data and statistical analysis

Data was not obtained for either variable during the active session of one participant due to a computer error. In addition, mean reaction times were not available for several participants who did not reject any unfair UG offers in either the sham or active condition. In total, data from 34 (sham) and 33 (active) participants was available for the number of unfair offer rejections variable, and from 25 (for both sham and active) participants for the reaction time of unfair offer rejections.

No outliers were identified in the final data set. Normality was assessed using the Kolmogorov–Smirnov test as well as through visual inspection of skewness and kurtosis values. Both the response data and response reaction time data were non-normally distributed and so non-parametric tests were used.

To test whether anodal stimulation to the rTPJ increased pro-social decision-making, Wilcoxon Signed Ranks test was first used to determine whether a difference existed in the number of rejected unfair offers between conditions. Following this, related samples non-parametric tests were applied to the response reaction time data. In the setting of apparent null results, Bayesian analyses were then performed.

Results were primarily analyzed using SPSS Statistics software (Version 22.0). In all cases, *p* < 0.05 were to be considered significant. Bayesian tests were later performed with JASP software (Version 0.8.6), with Bayes factors >1 interpreted as confirmation of the null hypothesis.

## Results

Means and standard deviations for the number and response reaction time of unfair offer rejections are presented in Table 1.

**Table 1:** Descriptive data for number and reaction time of unfair offer rejections in sham vs. active tDCS.

	**Sham**			**Active**		
	***n***	**Mean**	**SD**	***n***	**Mean**	**SD**
#	34	24.18	19.91	33	26.57	19.66
RT	25	1285.43	443.60	25	1221.34	397.68

**Figure 4:**
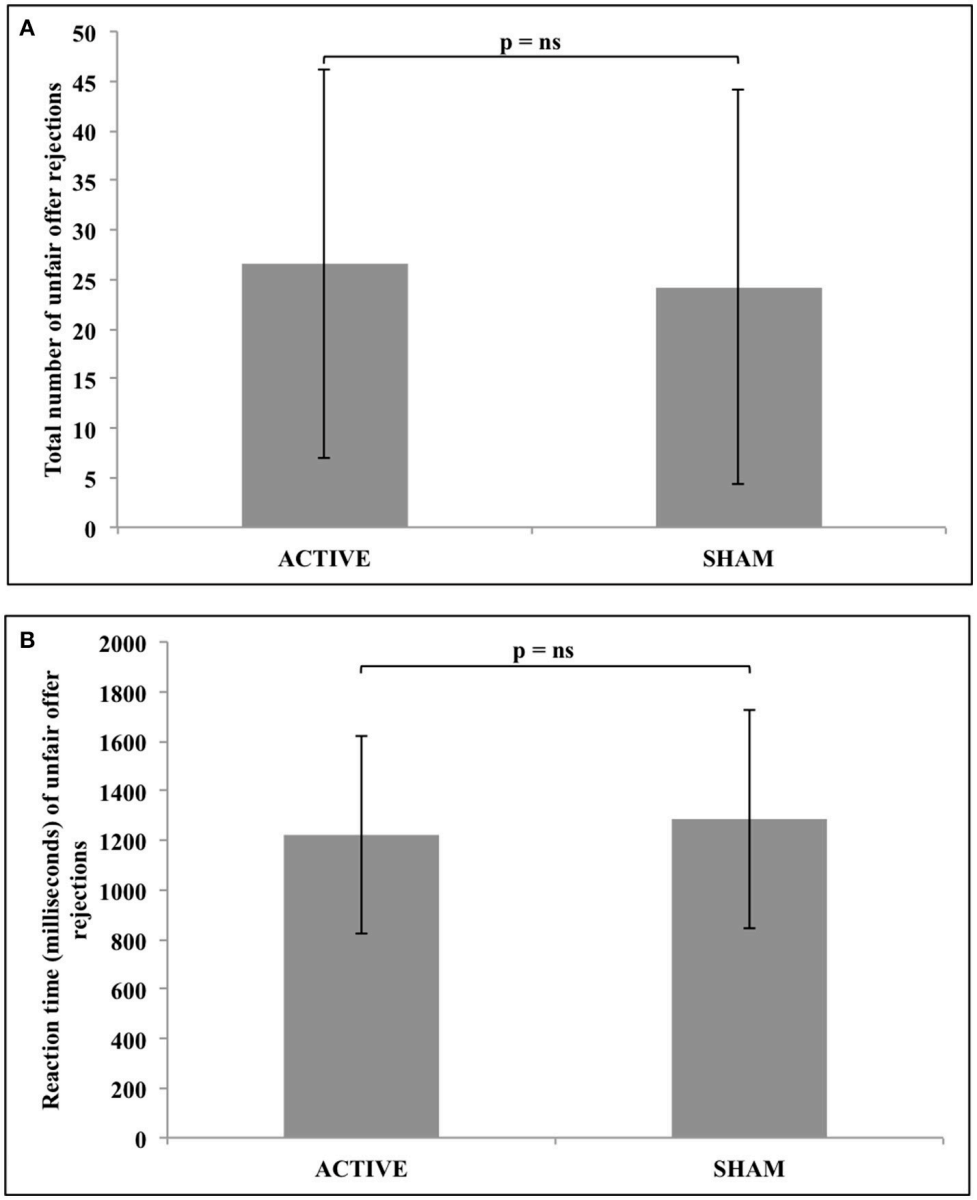
No significant difference in either **(A)** the total number of unfair offer rejections, or **(B)** the reaction time of unfair offer rejections following active vs. sham tDCS to the rTPJ.

Statistical analysis revealed no significant difference in either the total number (*z* = −1.031, *n* = 34, *p* = 0.303), or response reaction time (*z* = −0.608, *n* = 25, *p* = 0.543) of unfair offer rejections following active, compared to sham stimulation. These results are represented in Figure 4.

Bayesian analyses were then used to compare active and sham groups regarding the total number (BF_01_ = 3.566, error = 1.031e-6) and response reaction time (BF_01_ = 4.121, error = 0.035) data, confirming that the findings most likely represented a null effect from stimulation.

## Discussion

### Interpretation of results

The present study investigated the effects of anodal tDCS to the rTPJ on social decision-making. Our analysis revealed no significant difference in either the total number or response reaction time of unfair offer rejections made following active compared to sham stimulation. One could therefore conclude that the rTPJ is not involved in social decision-making when playing the UG, yet this notion seems inconsistent with numerous previous neuroimaging studies demonstrating heightened rTPJ activity during UG gameplay (Rilling et al., 2004; Halko et al., 2009; Guo et al., 2013; Van Den Bos et al., 2014). One potential explanation is that the rTPJ actively contributes to the mentalizing process when playing the UG (in determining an opponent's intentions as fair or unfair), without necessarily exerting full control over decision-making itself (and thus the number and speed of unfair offer rejections). This theory is in keeping with previous research stressing the complex neurobiology of social decision-making (Jeurissen et al., 2014), and its irreducibility to any one single element of social cognition (Frith and Singer, 2008). Indeed, in addition to social motivations possibly mediated by the rTPJ, numerous other brain regions and cognitive processes are thought to play a role in UG decision-making. For example, the AI may contribute negative emotional responses to unfair offers, and the DLPFC appears to exert executive control over an economically irrational impulse to reject such proposals (Sanfey et al., 2003).

Alternatively, the absence of a significant finding may reflect methodological considerations specific to tDCS, such as the strength, location, or duration of stimulation delivered. Indeed, these are common criticisms of the current tDCS literature, and the vast differences between research protocols have made results difficult to interpret and generalize. For example, tDCS studies using the 10/20 EEG system to locate the rTPJ have targeted vastly inconsistent sites (Donaldson et al., 2015), a concern that is heightened when considering potential inter-individual variability in the structure, size and location of the rTPJ, as well as the diffuse nature of stimulation effects. Similar discrepancies occur between studies with regards to the intensity of stimulation used, the presence or absence of a sham condition, and the washout period employed (Donaldson et al., 2015). Contrastingly, TMS (which has clearly established standard protocols and is able to target the rTPJ more focally) has been used successfully to modulate mentalizing and related socio-cognitive processes through rTPJ stimulation (Costa et al., 2008; Young et al., 2010; Giardina et al., 2011; Baumgartner et al., 2013; Jeurissen et al., 2014; Kelly et al., 2014; Bardi et al., 2017).

Finally, it is possible that the negative findings in this study can be traced back to problems with the social decision-making task itself. That is to say, maybe the UG was not specific enough to demonstrate the expected pro-social effects of rTPJ stimulation. Evidencing this, a 2013 NIBS study using a similar task from Game Theory found that parochial punishment of social norm defectors was decreased following rTPJ inhibition using TMS (Baumgartner et al., 2013). However, this effect was found to be mediated by retaliation motives, rather than desire to encourage normative behavior in others. The tension between self and fairness motives in the UG has been previously explored, and it appears that refusal of an unfair offer can indeed result from emotional resentment caused by poor treatment on a personal level, rather than pro-social intentions (Corradi-Dell'Acqua et al., 2013). In this setting, it is possible that participants in our study chose to punish their opponents out of anger and spite, rather than justice, in which case their responses might not have represented altruistic punishment (and thus pro-social behavior) at all. This may have been addressed in the current study by surveying responders about their specific motives in punishing unfair proposers.

### Limitations

There are some limitations to this research. As with many tDCS studies, the sample size was relatively small and results should be interpreted with appropriate care. In terms of the stimulation protocol, tDCS typically delivers diffuse stimulation, sensitive but not specific to the targeted region. This could be addressed through use of smaller electrodes (Nitsche et al., 2007) or with high-definition tDCS (Edwards et al., 2013). As for participant factors, inter-individual traits (including age, gender, anatomical, psychological, personality, and disease) could all contribute to variability of tDCS response (Dayan et al., 2013), and were not assessed for. Similarly, future research with a larger sample size may wish to explore individual variability in response to the UG, as this may affect whether tDCS modulates response.

Regarding the UG itself, whilst efforts were made to authenticate the social nature of the decision-making task, the anonymity of game partners may have raised participants' suspicion about whether they were genuinely interacting with other people (vs. a computer), in turn affecting the task's validity as a marker of social cognition and social decision-making (Frohlich et al., 2001; Sanfey et al., 2003; Lee and Harris, 2013). This may have been addressed by surveying participants as to whether they believed the cover story provided, or by making improvements to the task itself. Examples from previous literature include displaying opponents' (real or feigned) names and photographs instead of depersonalized identification numbers (Sanfey et al., 2003), simulating an internet-based opponent-matching system (Fitzgibbon et al., 2017), or arranging legitimate simultaneous group gameplay in a physical (Knoch et al., 2008) or online (Ciampaglia et al., 2014) setting. Interestingly, previous studies have demonstrated that people may reject unfair UG offers even when they know they are playing against a computer, and variously more (Torta et al., 2013), or less (Sanfey et al., 2003) often than when playing against human opponents. Such a factor further complicates interpretation of results.

### Implications and future directions

The current study did not identify a direct link between rTPJ function and social decision-making. Future research could incorporate optimized tDCS protocols with targeted, realistic social decision-making tasks in an attempt to yield significant results. Notably, this research identified complexities surrounding the connection between social cognition and social decision-making, particularly in an experimental setting.

Ultimately, further research in this field will help to advance understanding of the social brain, and of the neurobiology of conditions marked by abnormal social decision-making. Eventually, tDCS and related brain stimulation techniques may be trialed as novel treatment options for social symptomatology, and the earliest examples of this research show some promise. For example, a study that applied repetitive TMS to bilateral dorsomedial prefrontal cortices yielded a reduction in social relating impairment and social anxiety in individuals with Autism Spectrum Disorder (Enticott et al., 2014). Another group found that anodal tDCS administered over the bilateral DLPFC improved emotion identification in a social cognitive task in participants with schizophrenia (Rassovsky et al., 2015).

However, as clinical applications for noninvasive brain stimulation are increasingly developed and approved for use, it is of urgent importance to strengthen tDCS methodology and better develop the tools we use to assess social decision-making.

## Conclusion

This study applied anodal tDCS to the rTPJ in an attempt to modify social decision-making. Overall, there was found to be no significant difference in either the total number or reaction time of unfair offer rejections in the UG following active compared to sham tDCS.

This study highlights methodological issues in tDCS studies of the rTPJ, particularly as regards stimulation site and intensity, as well as task specificity. Moving forwards, optimized and standardized tDCS protocols should be developed to clarify and strengthen results, alongside rigorously tested social decision-making tasks.

## Author contributions

BF, KH, and PF conceived and supervised the study. BF, KH, and PH designed the experiments. LB-W carried out the experiments. LB-W, BF, and KH wrote the manuscript. All authors approved the final version of the submitted manuscript.

### Conflict of interest statement

PF has received equipment for research from MagVenture A/S, Medtronic Ltd., Neuronetics and Brainsway Ltd. and funding for research from Neuronetics. He is on scientific advisory boards for Bionomics Ltd. and LivaNova and is a founder of TMS Clinics Australia. The other authors declare that the research was conducted in the absence of any commercial or financial relationships that could be construed as a potential conflict of interest.
